# Genetic dysregulation of endothelin-1 is implicated in coronary microvascular dysfunction

**DOI:** 10.1093/eurheartj/ehz915

**Published:** 2020-01-23

**Authors:** Thomas J Ford, David Corcoran, Sandosh Padmanabhan, Alisha Aman, Paul Rocchiccioli, Richard Good, Margaret McEntegart, Janet J Maguire, Stuart Watkins, Hany Eteiba, Aadil Shaukat, Mitchell Lindsay, Keith Robertson, Stuart Hood, Ross McGeoch, Robert McDade, Eric Yii, Naveed Sattar, Li-Yueh Hsu, Andrew E Arai, Keith G Oldroyd, Rhian M Touyz, Anthony P Davenport, Colin Berry

**Affiliations:** 1 British Heart Foundation Glasgow Cardiovascular Research Centre, Institute of Cardiovascular and Medical Sciences, University of Glasgow, Glasgow G12 9DH, UK; 2 Department of Cardiology, Gosford Hospital, NSW, Australia; 3 Faculty of Medicine, University of Newcastle, NSW, Australia; 4 West of Scotland Heart and Lung Centre, Golden Jubilee National Hospital, Clydebank G81 4DY, UK; 5 Experimental Medicine and Immunotherapeutics, University of Cambridge, Level 6, Addenbrooke's Centre for Clinical Investigation (ACCI), Box 110, Addenbrooke's Hospital, Cambridge CB2 0QQ, UK; 6 Laboratory for Advanced Cardiovascular Imaging, National Heart, Lung, and Blood Institute, National Institutes of Health, Bethesda, MD, USA

**Keywords:** Endothelin-1, Single-nucleotide polymorphism, Stable angina pectoris, Coronary microvascular dysfunction, Microvascular angina, Precision medicine

## Abstract

**Aims:**

Endothelin-1 (ET-1) is a potent vasoconstrictor peptide linked to vascular diseases through a common intronic gene enhancer [(rs9349379-G allele), chromosome 6 (PHACTR1/EDN1)]. We performed a multimodality investigation into the role of ET-1 and this gene variant in the pathogenesis of coronary microvascular dysfunction (CMD) in patients with symptoms and/or signs of ischaemia but no obstructive coronary artery disease (CAD).

**Methods and results:**

Three hundred and ninety-one patients with angina were enrolled. Of these, 206 (53%) with obstructive CAD were excluded leaving 185 (47%) eligible. One hundred and nine (72%) of 151 subjects who underwent invasive testing had objective evidence of CMD (COVADIS criteria). rs9349379-G allele frequency was greater than in contemporary reference genome bank control subjects [allele frequency 46% (129/280 alleles) vs. 39% (5551/14380); *P* = 0.013]. The G allele was associated with higher plasma serum ET-1 [least squares mean 1.59 pg/mL vs. 1.28 pg/mL; 95% confidence interval (CI) 0.10–0.53; *P* = 0.005]. Patients with rs9349379-G allele had over double the odds of CMD [odds ratio (OR) 2.33, 95% CI 1.10–4.96; *P* = 0.027]. Multimodality non-invasive testing confirmed the G allele was associated with linked impairments in myocardial perfusion on stress cardiac magnetic resonance imaging at 1.5 T (*N* = 107; GG 56%, AG 43%, AA 31%, *P* = 0.042) and exercise testing (*N* = 87; −3.0 units in Duke Exercise Treadmill Score; −5.8 to −0.1; *P* = 0.045). Endothelin-1 related vascular mechanisms were assessed *ex vivo* using wire myography with endothelin A receptor (ET_A_) antagonists including zibotentan. Subjects with rs9349379-G allele had preserved peripheral small vessel reactivity to ET-1 with high affinity of ET_A_ antagonists. Zibotentan reversed ET-1-induced vasoconstriction independently of G allele status.

**Conclusion:**

We identify a novel genetic risk locus for CMD. These findings implicate ET-1 dysregulation and support the possibility of precision medicine using genetics to target oral ET_A_ antagonist therapy in patients with microvascular angina.

**Trial registration:**

ClinicalTrials.gov: NCT03193294.

**Figure:**
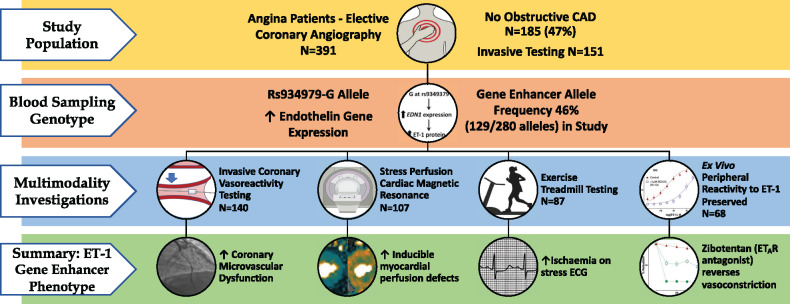

## Introduction

The coronary microcirculation has been implicated in the pathogenesis of angina for over 50 years, however, disease mechanisms remain incompletely understood.^1^ Coronary microvascular dysfunction (CMD) is associated with adverse outcomes in angina and a plethora of other cardiovascular disorders.^2–5^ Standardized diagnostic criteria for microvascular dysfunction^6^ underpin recent studies which have identified the disease prevalence affecting two-thirds of angina patients without obstructive epicardial coronary artery disease (CAD).^7–10^ These patients present a diagnostic and therapeutic challenge with up to one in four experiencing a major adverse cardiac event after 5 years of follow-up.^11^^,^^12^ The syndrome of ischaemia and no obstructive CAD (INOCA) is particularly important in women,^13^ whose elevated cardiac risk is mostly driven by impaired coronary flow reserve (CFR) (and not obstructive coronary disease).^11^

Endothelin-1 (ET-1) is a highly potent endogenous vasoconstrictor of human coronary arteries^14^ and has been implicated in the pathogenesis of microvascular dysfunction.^15^^,^^16^ Endothelin-1-mediated activation of the G protein-coupled endothelin A (ET_A_) receptor on vascular smooth muscle cells induces endothelial dysfunction, inflammation, and vasoproliferative effects. Circulating concentrations of serum ET-1 are inversely associated with coronary flow responses in patients with CMD.^14^^,^^16^ Recently, a common (39%) genetic locus in chromosome 6p24 (PHACTR1/EDN1) has been shown to be a distal regulator of endothelin gene expression.^17^ The allele, rs9349379-G, is associated with an increased risk for atherosclerotic epicardial CAD and myocardial infarction.^18^ This functional single-nucleotide polymorphism (SNP: rs9349379-G) is associated with increased endothelin gene expression resulting in a lifetime’s exposure of at least 20% higher ET-1 precursor levels in the plasma.^17^ Endothelin-1 dysregulation is implicated in coronary vascular disease, however, the role of rs9349379 in the pathogenesis of CMD has not been examined.

We investigated the association, if any, of the rs9349379-G allele with CMD in angina patients undergoing invasive coronary function testing. Our secondary objectives were to investigate whether the G allele associates with non-invasive parameters of myocardial ischaemia. Our final objective was to examine vascular mechanisms using isometric tension recordings in small peripheral resistance vessels isolated from patients according to genotype. We evaluated ET_A_ receptor-mediated vasoconstriction in subjects according to rs9349379-G allele status. These included zibotentan, an ET_A_ receptor-selective antagonist, which is available for repurposing following neutral results in phase 3 oncology trials.

## Methods

### Study population

We prospectively enrolled patients with stable angina. We screened elective adult referrals to two hospitals serving a population of ∼2.5 million in the West of Scotland. Patients were scheduled to undergo clinically indicated invasive coronary angiography for the investigation of suspected CAD. The participants were enrolled into the Coronary Microvascular Angina (CorMicA) study (ClinicalTrials.gov: NCT03193294), which was a randomized, controlled, strategy trial of stratified medicine in angina patients without obstructive CAD.^19^ Rose-Angina questionnaire was administered on the day of the angiogram and only patients with definite or possible angina were eligible to participate.^20^ Exclusion criteria included a non-coronary indication for invasive angiography, e.g. valve disease, severe renal dysfunction (glomerular filtration rate < 30 mL/min), inability to give informed consent and obstructive coronary disease determined during invasive coronary angiography [≥50% diameter stenosis and/or fractional flow reserve (FFR) ≤ 0.80]. All coronary vasodilating drugs were discontinued at least 24 h before the procedure. Pooled control genotype frequencies were ascertained from a contemporary medical genome reference cohort.^21^

### Definitions: coronary microvascular dysfunction

We defined CMD using invasive coronary function testing and the Coronary Vasomotion Disorders International Study Group (COVADIS) diagnostic criteria.^20^ These physiological criteria included an abnormal response to adenosine [raised index of microcirculatory resistance (IMR) (≥25) and/or abnormal CFR (<2.0)]. In addition, CMD also included subjects with microvascular spasm during acetylcholine (ACh) provocation [reproduction of angina symptoms, ischaemic electrocardiogram changes (≥1 mm ST-segment deviation), but <90% epicardial spasm during ACh testing].^22^ Coronary microvascular dysfunction is frequently associated with epicardial vasospasm and hence patients with abnormal vasoreactivity during adenosine assessment (abnormal IMR and/or CFR) *and* coexistent epicardial vasospasm during ACh provocation were included within the CMD group. Fractional flow reserve was measured to rule-out flow limiting CAD as an alternative explanation for myocardial ischaemia (INOCA subjects had an FFR >0.80 in target artery).

### Measurement of coronary vascular function *in vivo*

We used an interventional diagnostic procedure (IDP) that combined guidewire-based direct measurement of coronary vascular function followed by pharmacological vasoreactivity testing. Specifically, the IDP included a guidewire-based measurement of coronary vascular function [FFR, CFR and IMR] followed by pharmacological vasoreactivity testing with ACh and glyceryl trinitrate (GTN) and has been previously described.^19^^,^^23^

In brief, an intravenous infusion of adenosine (140 μg/kg/min) was administered via a large peripheral vein to induce steady-state maximal hyperaemia for a period of at least 90 s with a target time of 180 s. A pressure–temperature sensitive guidewire was placed into the distal third of a major epicardial coronary artery (typically the left anterior descending). The myocardial FFR was calculated by the ratio of mean distal coronary pressure to mean aortic pressure during maximal hyperaemia. A FFR ≤0.80 was taken as abnormal and indicative of flow-limiting CAD.^24^ Coronary flow reserve was calculated using thermodilution as resting mean transit time divided by hyperaemic mean transit time.^25^ A CFR <2.0 was defined as abnormal representing impaired vasodilator reserve.^26^ The IMR was calculated as the product of mean hyperaemic transit time and mean distal coronary pressure at hyperaemia.^27^ An IMR ≥25 was defined as abnormal and indicative of increased microcirculatory resistance.^28^ These invasive parameters were simultaneously derived in real time using dedicated software (Coroventis, Uppsala, Sweden). We assessed endothelium-dependent coronary vasomotor function using intracoronary infusions of ACh via the guiding catheter at concentrations of 0.182, 1.82, and 18.2 µg/mL (10^−6^, 10^−5^, and 10^−4 ^ mol/L, respectively) at 1 mL/min for 2 min via a mechanical infusion pump.^29^ Patients who had CMD (e.g. abnormal CFR and/or IMR) but co-existent epicardial vasospasm during ACh bolus (100 μg bolus of ACh; 5.5 mL of 10^−4 ^ mol/L over 20 s) were considered in the CMD group.^30^ In order to assess non-endothelial dependent vasodilatation, 300 µg of GTN was administered by manual intracoronary bolus injection. Detailed methods are reported in the Supplementary material online, *Appendix*.

### Blood and tissue analysis

Serum ET-1 was determined using blood obtained on the day of coronary function testing (Quantikine ^®^ ELISA, R&D Systems^®^ Europe, Abington, UK). Blood was obtained from participants following an overnight fast in a recumbent position.

*Ex vivo* pharmacological assessment of peripheral vascular function was performed on patients who volunteered to undergo a gluteal skin fat biopsy within 4 weeks of the invasive coronary function assessment. The biopsy was obtained under sterile conditions using local anaesthesia with lidocaine (2%). Small peripheral resistance vessels (<400 µm) were carefully dissected from fresh biopsies using a light microscope. About 2 mm length vessels were mounted on 40-μm stainless steel wires for isometric myography in multi-channel myograph chambers (DMT, Denmark) filled with physiological saline solution. Isometric tension recordings followed-on directly using the technique of wire myography to study small peripheral resistance arteries with paired cumulative concentration response curves (CCRCs) to ET-1 in the presence or absence of an ET_A_ receptor antagonist, either BQ123 or zibotentan (AstraZeneca, UK; Open Innovation). This vascular biology sub-study was an extension of our work in INOCA subjects that was previously published in this journal.^31^ The detailed methods are described in the Supplementary material online, *Appendix*. The peripheral vascular sensitivity to ET-1 (pEC_50_) and maximum vasoconstriction to ET-1 (*E*_max_) were determined.

For the antagonist studies, the affinity (K_B_) of BQ123 was first determined in paired vessels from individuals and calculated using Schild regression. The pK_B_ (−log_10_ K_B_) values were compared between each genotype as an indicator of whether or not patients of different genotypes are likely to respond equally well to an ET_A_ antagonist used clinically. A final series of experiments involved paired vessel experiments using ET-1 CCRCs in the presence and absence of a highly selective ET_A_ receptor antagonist, zibotentan to determine a pK_B_ value and assess whether zibotentan could reverse an established ET-1-mediated vessel constriction.

### Cardiac magnetic resonance imaging and ischaemia testing protocol

Patients were prospectively invited to undergo quantitative perfusion cardiac magnetic resonance (CMR) imaging at 1.5 T using pharmacological stress testing with intravenous adenosine (140 µg/kg/min) within 6 weeks of the index coronary angiogram. CMR studies were performed using a standardized CMR protocol (Siemens MAGNETOM Avanto, Erlangen, Germany). The CMR scans were interpreted by two experienced observers (D.C., C.B.) with Level III accreditation of the European Association of Cardiovascular Imaging (EACVI), blind to diagnostic findings and genotype. The raw stress and rest perfusion images were qualitatively assessed for inducible or fixed perfusion defects. The perfusion was classified as either normal, abnormal, or equivocal. If a perfusion defect was present, it was reported as having an epicardial, microvascular or equivocal pattern. Perfusion defects were then reported on a segmental basis according to the American Heart Association 16-segment model^32^ and were classified according to the transmurality of the perfusion defect (<50% or >50%), and the number of segments with qualitatively abnormal perfusion was defined. Dark rim artefact was adjudicated based on standardized criteria.^33^

The first-pass perfusion images were then post-processed to derive quantitative pixel perfusion maps to derive absolute myocardial blood flow and myocardial perfusion reserve (MPR) (further detail in Supplementary material online).^34^

Treadmill exercise stress electrocardiography using the Bruce protocol was analysed from the subgroup of patients who had been pre-selected for this procedure on clinical grounds prior to invasive coronary angiography. We used the Duke treadmill score (DTS) which is a validated metric with established prognostic cardiovascular utility.^35^ The exercise treadmill test analysis included (i) exercise duration and (ii) the DTS^36^ by a cardiology researcher (EY) blinded to genotype and invasive physiology. The DTS is based on the occurrence of angina during treadmill exercise testing, ST-segment depression during the test and peak exercise duration (or metabolic equivalent of task achieved). Specifically, the DTS equals the maximum exercise time in minutes − (5 × the maximal net ST-segment deviation in mm during or after exercise) − (4 × the treadmill angina index (where 0 = no angina, 1 = non-limiting angina, 2 = exercise limiting angina).

All subjects were asked to abstain from caffeine-containing beverages or foodstuffs for 24 h, and vasoactive medications for 48 h prior to the CMR examination. All scan acquisitions were spatially co-registered. All CMR analyses were performed by a blinded analyst with Level 3 EACVI accreditation.

### Statistical analysis

The main hypothesis in our study was that regulation of ET-1 gene expression reflected by the presence of the intronic ET-1 gene enhancer, rs9349379-G, associates with invasive tests of CMD. We tested the association of genotype (SNP rs9349379 G-A allele status) with CMD on invasive coronary vasoreactivity testing by calculating the odds ratio (OR) and its 95% confidence intervals (CIs). Multivariable logistic regression was used to determine whether genotype was independently associated with CMD (as defined by abnormal response to intracoronary ACh and/or systemic adenosine) adjusting for overall cardiac risk (ASSIGN score) including previous cardiac events.^37^

Categorical data are presented as percentages and continuous parameters are shown as means with standard deviation values or medians with interquartile ranges. For secondary analyses, subjects were divided into three genotype groups. Kruskal–Wallis test was used to test whether distribution of non-parametric variables is the same between the groups. Subgroup analysis of A vs. G genotypes was determined a priori to evaluate any differences between the two most differentiated groups. The least squares (LS) mean of serum ET-1 levels was compared between the groups derived using analysis of co-variance with serum ET-1 as dependent variable and adjusted for age, sex, body mass index, genotype and cardiovascular risk as covariates and possible confounders. Linear associations with invasive and non-invasive metrics of microvascular disease were performed by analysis of variance (ANOVA) with *P* for linear trend for continuous parameters and χ^2^ test with *P* for linear-by-linear test for categorical variables. Statistical analyses were performed with Prism 7.0 (GraphPad, La Jolla, CA, USA) and SPSS 25.0 (SPSS, Chicago, IL, USA).

## Results

We prospectively enrolled 391 patients with angina between 25 November 2016 and 11 December 2017 at two hospitals serving a population of ∼2.5 million in the West of Scotland (CorMicA: ClinicalTrials.gov NCT03193294).^19^ Invasive coronary angiography revealed obstructive disease in 206 (53.7%) participants who were then excluded from further study. One hundred and fifty-one participants with no obstructive coronary disease continued in the study (*Take home figure*, *Table 1*). Evidence of CMD was found in 109 (72%) of 151 subjects undergoing invasive coronary vasoreactivity testing (*Table 2*). An overview of the study and investigations is illustrated in *Take home figure*. Genetic analysis was completed in 140 subjects (93%) using baseline venous blood samples. The mean age of patients in this analysis 61.1 ± 10.1 years. There was a predominance of women [103 (74%)] and the estimated 10-year risk of cardiovascular events (ASSIGN) was appreciable at 25% (±20).

**Figure 1:**
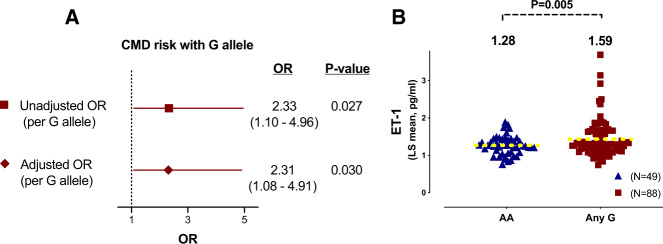
Detrimental effects of rs9349379-G allele on coronary microvascular function and endothelin-1. (*A*) Patients with G allele were over twice as likely to have underlying microvascular dysfunction (odds ratio per G allele 2.33, 95% confidence interval 1.10–4.96; *P* = 0.027) Even after adjustment for other risk factors the G allele was predictive of microvascular disease (odds ratio 2.31; 95% confidence interval 1.0–4.91). This finding supports a detrimental impact on the coronary microcirculation of a lifetime of increased endothelin gene expression. (*B*) In a multivariable regression model adjusting for baseline group differences, patients with rs9349379-G allele had higher plasma endothelin-1 (least squares mean 1.59 pg/mL vs. 1.28 pg/mL; 95% confidence interval 0.10–0.53; *P* = 0.005).

**Table 1:** Baseline demographics by genotype

	SNP (rs9349379) genotype (*n* = 140)			*P*-value^a^
	AA (*N* = 50)	AG (*N* = 51)	GG (*N* = 39)	
Clinical features				
Age (years)	60.6 (±11)	61.1 (±10)	61.6 (±10)	0.649
Female	36 (72%)	36 (71%)	31 (80%)	0.607
ASSIGN score^b^	24 (±21)	27 (±23)	25 (±19)	0.811
Dyslipidaemia	12 (24%)	10 (20%)	8 (21%)	0.671
Hypertension	30 (60%)	32 (63%)	27 (69%)	0.382
Previous cardiovascular event^c^	10 (20%)	10 (20%)	13 (33%)	0.239
Diabetic	9 (18%)	11 (22%)	6 (15%)	0.794
Smoker	6 (12%)	8 (16%)	9 (23%)	0.169
Family history	17 (34%)	13 (26%)	13 (33%)	0.886
Peripheral vascular disease	2 (4%)	3 (6%)	2 (5%)	0.789
Atrial fibrillation	5 (10%)	4 (8%)	1 (3%)	0.195
Pulse (rate/min)	69 (±11)	67 (±11)	71 (±11)	0.697
Systolic blood pressure (mmHg)	138 (±22)	136 (±31)	138 (±25)	0.951
Diastolic blood pressure (mmHg)	73 (±11)	74 (±15)	70 (±12)	0.260
Body mass index (kg/m^2^)	30.4 (±8)	30.4 (±6)	29.4 (±7)	0.515
Laboratory investigations				
Cholesterol (mmol/L)	3.5 (±1)	3.5 (±1)	3.6 (±1)	0.904
Glucose (mmol/L)	4.6 (±1)	5.0 (±2)	4.7 (±2)	0.774
C-reactive protein (mg/L)	3.2 (±5)	3.2 (±5)	3.1 (±4)	0.920
N-terminal brain natriuretic peptide (pg/mL)	140 (±187)	157 (±197)	135 (±153)	0.937
Endothelin-1 (pg/mL)^d^	1.27 (0.42)	1.41 (0.63)	1.46 (0.56)	0.097

Data are expressed as mean (standard deviation) or number (%).

ACE-I, angiotensin converting enzyme inhibitor; ACh, acetylcholine; BMI, body mass index; CCB, calcium channel blocker; CFR, coronary flow reserve; FFR, fractional flow reserve; IMR, index of microcirculatory resistance; LVEDP, left ventricular end-diastolic pressure; MI, myocardial infarction.

a *P*-value represents between group ANOVA for linear trend (continuous data) or Pearson χ^2^ test for linear trend (categorical data) or Kruskal–Wallis testing probability that the distribution of non-parametric variables are the same across the groups.

b ASSIGN risk—predicted 10-year risk of cardiovascular event.

c Previous myocardial infarction or cerebrovascular event (including transient ischaemic attack).

d Endothelin-1 levels were available in 137 genotyped subjects with significance determined using one-way ANOVA (linear trend).

**Table 2:** Invasive coronary physiology and non-invasive stress testing

	SNP (rs9349379) genotype			*P*-value^a^
	AA (*N* = 50)	AG (*N* = 51)	GG (*N* = 39)	
Minor non-obstructive CAD^b^	25 (50%)	30 (59%)	24 (62%)	0.265
Coronary atheroma burden (Gensini score)^c^	0 (0.2)	2 (0.5)	1 (0.6)	0.037
Left ventricular end-diastolic pressure (mmHg)	10 (±4)	10 (±5)	9 (±3)	0.520
Fractional flow reserve (FFR)	0.88 (0.05)	0.88 (0.06)	0.88 (0.05)	0.977
Coronary microvascular dysfunction (any)	30 (60%)	38 (75%)	32 (82%)	0.021
Abnormal CFR (<2.0)	10 (20%)	18 (36%)	16 (41%)	0.030
Coronary flow reserve (CFR)	3.0 (2.1–3.7)	2.7 (1.8–3.5)	2.1 (1.7–3.2)	0.046
Abnormal IMR (≥25)	12 (24%)	17 (33%)	18 (46%)	0.029
Microcirculatory resistance (IMR)	18.9 (15.2–24.2)	18.6 (14.2–29.3)	22.1 (13.8–29.3)	0.879
Abnormal CFR or IMR	20 (40%)	26 (51%)	27 (69%)	0.007
Microvascular spasm (during acetylcholine)	15 (30%)	21 (42%)	12 (31%)	0.385
Exercise treadmill testing (*N* = 87)	28 (56%)	34 (67%)	25 (64%)	
Duration (s)	393 (±124)	352 (±157)	384 (±162)	0.827
METs	7.8 (±2.1)	7.4 (±2.6)	7.6 (±2.1)	0.786
Angina on treadmill	16 (59%)	23 (68%)	20 (87%)	0.036
Peak systolic blood pressure (mmHg)	178 (±30)	173 (±34)	182 (±25)	0.688
Duke Treadmill Score	−0.3 (±6.0)	−0.6 (±4.7)	−3.3 (±4.2)	0.045
Stress perfusion magnetic resonance imaging (*N* = 107)				
Inducible myocardial perfusion defect	11 (31%)	17 (43%)	18 (56%)	0.042
Inducible myocardial perfusion defect with CMD	4 (13%)	14 (37%)	15 (47%)	0.016
Myocardial perfusion reserve (global)	1.8 (±0.4)	1.7 (±0.4)	1.6 (±0.4)	0.154
Myocardial perfusion reserve (endocardium)	1.7 (±0.4)	1.6 (±0.4)	1.5 (±0.4)	0.162
Left ventricular end diastolic volume (indexed, mL/m^2^)	68.5 (±13.6)	70.1 (±13.2)	70.2 (±11.9)	0.591
Left ventricular end systolic volume (indexed, mL/m^2^)	23.4 (±6.0)	25.4 (±8.8)	23.1 (±5.8)	0.848
Left ventricular ejection fraction (%)	65.9 (±4.4)	64.5 (±6.5)	67.3 (±5.2)	0.321
Stroke volume (indexed, mL/m^2^)	45.0 (±8.8)	44.7 (±7.0)	47.1 (±8.2)	0.298
Left ventricular mass (indexed, mL/m^2^)	42.0 (±7.0)	42.3 (±8.1)	42.1 (±7.8)	0.924

Data are expressed as mean (±SD), median (IQR), or *N* (%).

CAD, coronary artery disease; CFR, coronary flow reserve; FFR, fractional flow reserve; LVEDP, left ventricular end-diastolic pressure; IMR, index of microcirculatory resistance; METS, metabolic equivalent of task.

a *P*-value represents between group ANOVA for linear trend (continuous data) or Pearson χ^2^ test for linear trend (categorical data), Kruskal–Wallis test of probability that the distribution of non-parametric variables are the same across the groups.

b Core-laboratory adjudication of any angiographic evidence of coronary atherosclerosis including any minimal angiographic luminal irregularity.

c Gensini angiographic score is a metric of angiographic disease severity incorporating lesion severity and location. Detailed MRI methodology available in Supplementary material online, *Appendix*.

**Figure 2:**
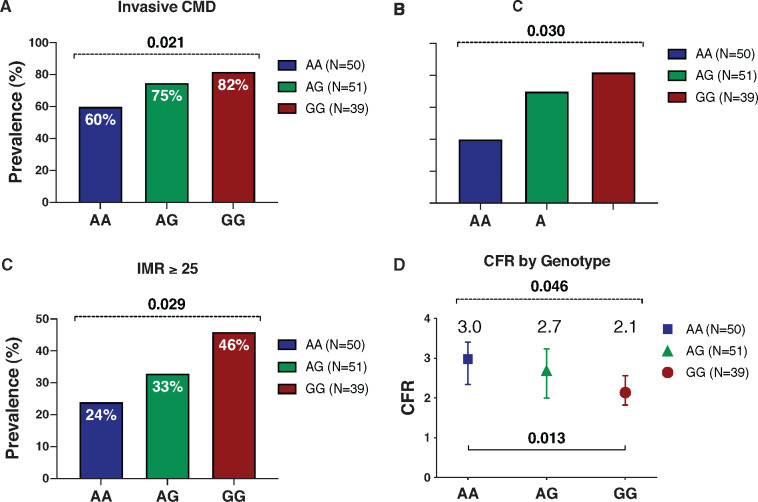
Genotype: phenotype association of G allele with invasive coronary microvascular dysfunction. (*A*–*C*) The prevalence of microvascular dysfunction detected during invasive coronary testing was associated with genotype status (AA 60%, AG 75%, GG 83%; *P* = 0.021). Presence of abnormal coronary flow reserve and microcirculatory resistance were linearly associated with each additional G allele. *P*-value represents Pearson χ^2^ test for linear trend (categorical data). (*D*) Coronary flow reserve was lower amongst subjects with two high-risk G alleles (rs9349379) consistent with detrimental effects of increased endothelin gene expression on the coronary microcirculation (Kruskal–Wallis between groups dotted line, *P* = 0.046). A priori subgroup analysis (AA vs. GG group—solid line) showed lower CFR in the GG group (*P* = 0.013). Data are median CFR plus error bars represent 95% confidence intervals for the median. *P* = 0.021, *P* = 0.030, *P* = 0.029 and *P* = 0.046.

The genotype distribution of rs9349379 was AA (*N* = 50, 36%), AG (*N* = 51, 36%), and GG (*N* = 39, 28%). This SNP did not fulfil Hardy Weinberg equilibrium (*P* = 0.0015) reflecting biologic ascertainment of genotypes. One hundred and forty subjects underwent genetic analysis for (rs9349379-G allele) with an allele frequency of 46% (129/280 alleles). The allele frequency was increased in our angina cohort compared to that of genome bank control subjects [rs9349379-G allele frequency 39% (5551/14380); χ^2^ = 6.15, *P* = 0.013].^21^ The rs9349379-G allele was associated with over double the odds of CMD (OR 2.33, 95% CI 1.10–4.96; *P* = 0.027; *Figure 1A*). Subjects with G allele had higher circulating serum ET-1 concentration (LS mean 1.59 pg/mL vs. 1.28 pg/mL; difference 0.31 pg/mL; 0.10–0.52; *P* = 0.005; *Figure 1B*). Each additional G allele was linearly associated with CMD on invasive interrogation (*Figure 2A*; *P* = 0.021). On multivariable analysis, the G allele remained associated with CMD (OR per G allele 2.31; 1.08–4.91; *P* = 0.030; Supplementary material online, *Table* *S1*).

**Figure 3:**
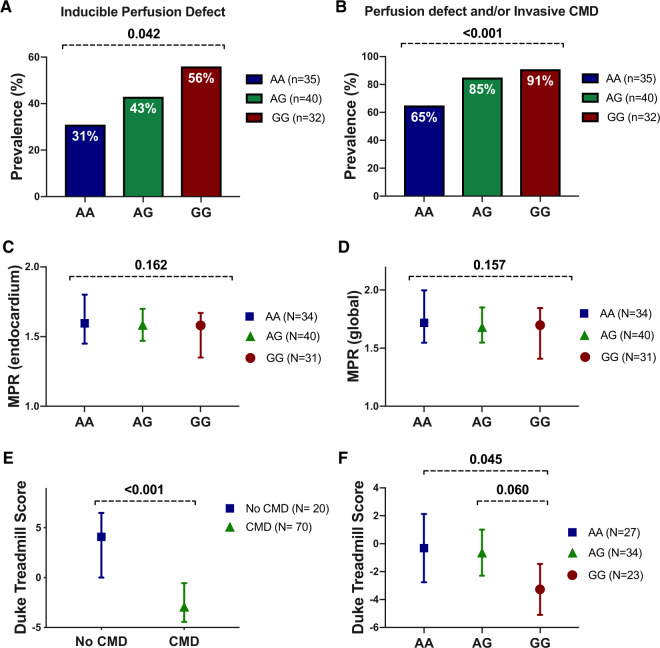
Genotype: phenotype association of G allele with non-invasive ischaemia testing. (*A*) Cardiovascular stress magnetic resonance imaging at 1.5 T (*N* = 107). There was a linear relationship between the G allele and presence of an inducible perfusion defect on cardiac magnetic resonance (χ^2^ test for linear trend *P* = 0.042). (*B*) The relationship was more robust when considering with invasive evidence of coronary microvascular dysfunction and/or inducible perfusion defect. Over 90% of GG subjects had at least one abnormality compared with only 65% of AA subjects (*P* < 0.001). (*C* and *D*) Myocardial perfusion reserve was numerically reduced in AG and GG subjects compared with AA subjects; however, this was not statistically significant (*P*-value represents analysis of variance test for trend). Error bars represent 95% confidence intervals for the mean. (*E*) Invasive evidence of microvascular dysfunction (defined by abnormal response to intracoronary acetylcholine and/or systemic adenosine) was functionally significant and associated with ischaemic burden on symptom limited exercise treadmill testing (coronary microvascular dysfunction −2.3 vs. no coronary microvascular dysfunction +3.5; difference −5.8 units; −8.2 to −3.3; *P* < 0.001). (*F*) Exercise treadmill testing (*n* = 84). There was a relationship between genotype group and worsening ischaemia on stress testing (analysis of variance *P*-trend = 0.045). The mean difference in ischaemia by Duke treadmill score between group GG and group AA was −3.0 units (95% confidence interval −5.8 to −0.1; *P* = 0.045). Error bars represent 95% confidence intervals for the mean.

Considering diagnostic subtypes of microvascular dysfunction, the vast majority had CMD during adenosine interrogation (73% abnormal CFR and/or IMR) and only 27% of the genotyped population had isolated microvascular spasm (isolated CMD to ACh only). There was a statistically significant relationship between genotype and CMD, as reflected by an impaired coronary vasodilator reserve (abnormal CFR: AA 20%, AG 35%, and GG 41%;*Figure 2B*; *P* = 0.030). A similar relationship was noted for prevalence of abnormal microvascular resistance in each genotype (abnormal IMR: AA 24%, AG 33%, and GG 46%; *Figure 2C*; *P* = 0.029). Coronary flow reserve decreased linearly with each additional rs9349379-G allele [AA 3.0 (2.1–3.7); AG 2.7 (1.8–3.5); GG 2.1 (1.7–3.2); overall *P* = 0.046; *Figure 2D*; *Table 2*]. The highest risk group (GG) had a significantly lower CFR than the AA group (median difference 0.84, 95% CI 0.1–1.1). The prevalence of abnormal invasive ACh response was not statistically different between the groups (any G allele 36% vs. no G allele 30%, *P* = 0.463). Patients with isolated CMD to ACh (microvascular spasm) had similar ET-1 levels to those without (1.33 ng/mL vs. 1.28 ng/mL; *P* = 0.769). The highest serum ET-1 levels were seen in subjects with concordant abnormalities in both CFR and IMR with linear stepwise reduction compared to those with only one index of CMD and lowest in those without any abnormalities [mean 1.67 ng/mL (both) vs. 1.39 ng/mL (one) vs. 1.31 ng/mL (none); *P* trend = 0.041].

The Gensini angiographic score reflecting the extent (or burden) of coronary atherosclerosis was higher in the rs9349379-GG group [median score 1.0 (0.0–6.0)] compared to the AA group [median score 0.0 (0.0–2.0); *P* = 0.037; *Table 2*]. As might be expected in this population of INOCA patients, the physiological burden of epicardial CAD was similar between the groups [myocardial FFR, AA 0.88 (±0.05); AG 0.88 (0.06); GG 0.88 (±0.05); *P* = 0.977].

One hundred and seven subjects underwent an adenosine stress perfusion cardiac magnetic resonance imaging (MRI) within 6 weeks of the invasive angiogram. Forty-six (43%) patients had evidence of a sub-endocardial circumferential abnormality of myocardial perfusion attributable to CMD (*Table 2*). The rs9349379-G allele was associated with abnormal myocardial perfusion disclosed by stress perfusion MRI (AA 31%, AG 43%, GG 56%; *P* = 0.042, *Figure 3A*). The association of genotype with CMD was more robust when considering subjects with either a circumferential subendocardial perfusion defect disclosed by MRI or invasive evidence of CMD (AA 65%, AG 85%, GG 91%; *P* < 0.001; *Figure 3B*). The absolute global and subendocardial perfusion reserve (MPR) was numerically lower with each G allele; however, the differences were not statistically significant (*Table 2*; *Figure 3C* and *D*).

**Figure 4:**
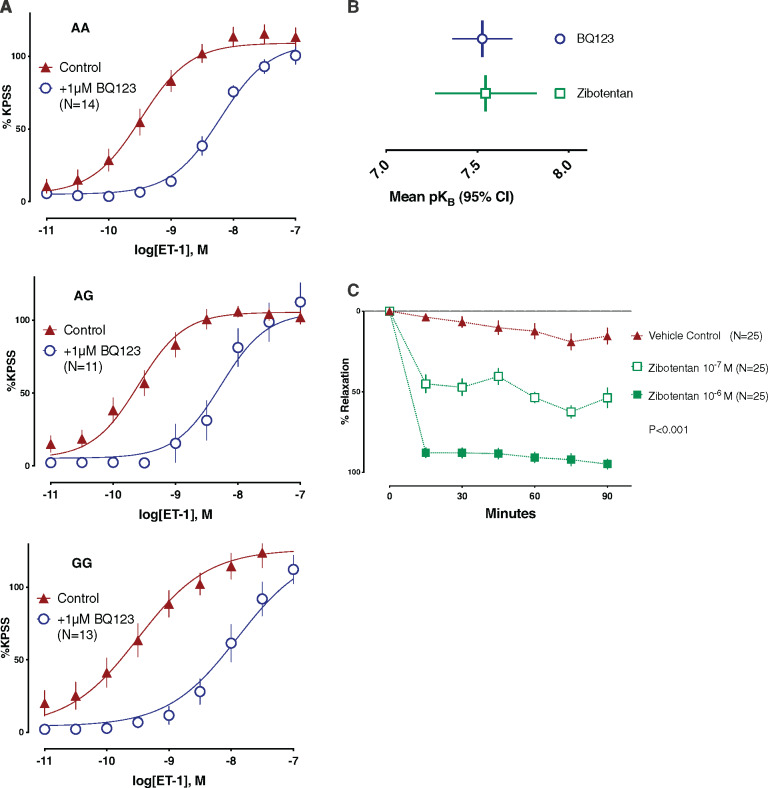
Endothelin-1 *ex vivo* vascular biology by genotype. (*A*) cumulative concentration response curve to endothelin-1 in the three groups in the presence and absence of ET_A_ antagonist BQ123 (*n* = 44). Similar antagonist potency (rightward curve shift) for each group suggesting firstly that the ET_A_ receptors are the dominant effectors of the endothelin-1 vasoconstrictor response and secondly that the ET_A_ receptor pathway is not down-regulated in spite of the elevated endothelin-1 gene expression and known increase in endothelin-1 activity in the G allele single-nucleotide polymorphism patients. (*B*) Antagonist potency of novel therapeutic oral ET_A_ receptor antagonist zibotentan [*N* = 8, mean 7.54 (95% confidence interval 7.27–7.82)] is similar to peptide antagonist BQ123 [*N* = 27, mean 7.53 (95% confidence interval 7.37–7.69)]. Higher pK_B_ represents a higher antagonist potency. (*C*) Zibotentan: reversal of established endothelin-1 vasoconstriction. Proof of concept dose-dependent reversal of potent and established endothelin-1-mediated peripheral arteriolar vasoconstriction. Crucially, the highest concentration tested which is also the plasma concentration achieved by a clinically relevant dose of 10 mg/day rapidly and fully reversed the established endothelin-1 constrictor response, indicative of efficacy in conditions of vasospasm. Comparison using ordinary two-way analysis of variance including time and dose both significant factors (*P* < 0.001 after adjustment for multiple testing).

We then assessed relationships between exercise treadmill testing, invasive measures of coronary vascular function and genotype. Ninety subjects prospectively completed exercise treadmill testing during standard care diagnostic work up prior to invasive coronary angiography, 84 of these subjects were included in the study with the remainder being excluded due to lack of genotype or exercise data. The mean exercise duration was 367 s (±156 s) and similar between the groups (*Table 2*). The mean DTS was −1.0 (±5.3) units. The presence of CMD was associated with reduced DTS (CMD −2.3 vs. no CMD +3.5; difference −5.8 units, 95% CI −8.2 to −3.3; *P* < 0.001; *Figure 3E*). Overall, there was a moderate inverse correlation between presence of CMD and the DTS (Spearman’s rho = −0.42; *P* < 0.001). Considering the cohort of 84 patients in whom genotype and DTS were both available, there was a lower DTS for each additional G allele consistent with increasing ischaemia with ET-1 gene enhancement. *A priori* analysis of high-risk subjects (homozygous for the minor G allele) compared to the AA group revealed a mean difference of −3.0 units in DTS (95% CI −5.8 to −0.1; *P* = 0.045) (*Figure 3F*). There was a modest correlation between the continuous DTS and genotype (Spearman’s rho −0.21; *P* = 0.055), that was not statistically significant. The angina index during exercise was linearly associated with G allele status (non-limiting or limiting angina AA 59% vs. AG 68% vs. GG 87%; *P* trend =0.036). The exercise time was not significantly lower amongst subjects with the G allele (365 vs. 392 s; *P* = 0.423).

Sixty-eight genotyped subjects agreed to participate in a vascular biology sub-study, providing written informed consent for a gluteal subcutaneous biopsy within 4 weeks of coronary angiography. Subjects who volunteered to have a biopsy were of similar age and cardiac risk to those who declined to participate in the sub-study [biopsy participants mean age 62 ± 9 years vs. 61 ± 11 years (*P* = 0.134), ASSIGN score 23% ± 18 vs. 28% ± 23 (*P* = 0.198)]. Forty-four (65%) of these patients had biopsies with a sufficient number of small arteries to undergo paired CCRCs to ET-1 in the presence and absence of an ET_A_ receptor antagonist, either BQ123 or zibotentan (ZD4054; AstraZeneca, Cambridge, UK). Grouping according to genotype (AA, *n* = 16; AG, *n* = 14; GG, *n* = 14) and vasodilator responses to ACh (ACh *E*_max_) were similar (*Table 3*). Similarly, vessels had similar potency for ET-1 (pEC_50_ AA 9.34, AG 9.45, and GG 9.32; *P* = 0.533) and maximum vasoconstriction to ET-1 (*E*_max_ AA 122.3%, AG 115.5%, GG 129.7%; *P* = 0.533; *Figure 4A*; *Table 3*).

**Figure 5:**
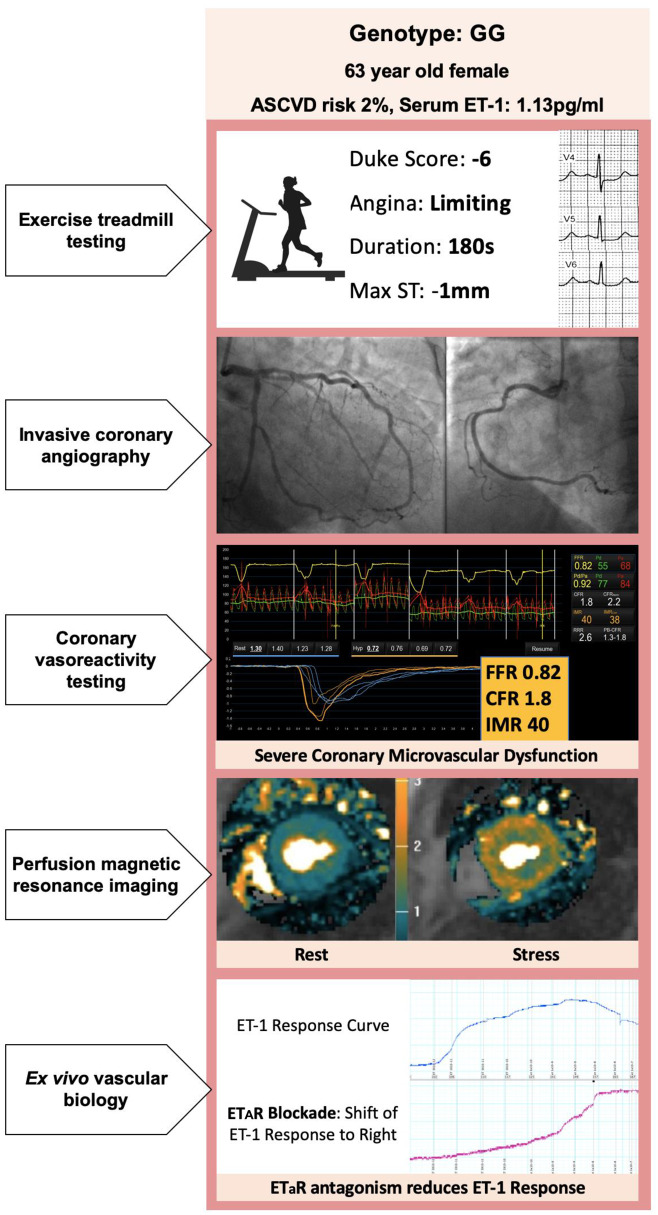
GG (high-risk endothelin-1 gene enhancer). Illustrative case from a patient with stable angina including representative images from invasive and non-invasive work up are shown in relation to clinical presentation and endothelin-1 enhancer genotype. Maximum ST represents the maximum planar or down sloping ST-segment depression during the exercise treadmill test. Invasive coronary angiography of both subjects is near identical showing only minimal luminal irregularities. White arrows represent subendocardial inducible ischaemic myocardium during adenosine stress magnetic resonance imaging in a patient with severe coronary microvascular dysfunction. *Ex vivo* vascular biology (bottom panel) shows typical endothelin-1-mediated vessel constriction during wire myography. Increasing vessel tension corresponds to the rising curve at each dose titration. A paired identical vessel experiment is performed after incubation with BQ123, an ET_A_ receptor antagonist. This curve is marked in blue, the curve of endothelin-1 response is shifted to the right indicating that the ET_A_ receptor mediates vasoconstriction. Despite the endothelin-1 gene enhancer, the GG subject does not appear to have ET_A_ receptor down-regulation with similar levels of antagonist potency. This supports that ET_A_ receptor antagonism in this group of patients may have therapeutic benefit. CFR, coronary flow reserve; ET_A_, endothelin A receptor; FFR, fractional flow reserve; IMR, index of microcirculatory resistance.

**Table 3:** Pathophysiology: vascular biology of ET-1

	SNP (rs9349379) genotype (*n* = 44)			*P*-value^a^
	AA (*N* = 16)	AG (*N* = 14)	GG (*N* = 14)	
Vessel diameter (um)	344 (±88)	342 (±89)	347 (±125)	0.851
Vessel length (mm)	1.85 (±0.12)	1.87 (±0.10)	1.82 (±0.11)	0.276
ACh *E*_max_ (%)	77.7 (52.9–97.8)	80.2 (59.9–97.6)	92.5 (57.8–99.1)	0.696
ACh pEC_50_	7.28 (6.88–7.82)	7.26 (6.82–8.00)	6.96 (6.84–7.44)	0.308
ET-1 *E*_max_ (%)	122.3 (115.7–134.7)	115.5 (107.5–125.2)	129.7 (115.8–151.2)	0.533
ET-1 pEC_50_	9.34 (9.15–9.52)	9.45 (9.24–9.67)	9.32 (8.96–9.69)	0.533
BQ123 pK_B_ (±SEM)	7.07 (±0.23)	7.79 (±0.35)	7.41 (±0.26)	0.209

Forty-four (65%) of 68 patients who underwent invasive biopsies had a sufficient number of small arteries to undergo paired cumulative concentration response curves (CCRCs) to ET-1 in the presence and absence of an ET_A_ receptor antagonist. Data are expressed as mean (±SD) or mean (95% CI for pooled best fit CCRC). CCRC, cumulative concentration response curves were drawn with best-fit derived values. pK_B_ data involved paired vessels undergoing ET-1 CCRC in the presence or absence of BQ123 ET_A_ receptor antagonist (available in 37 out of the 44 subjects: AA *N* = 14; AG *N* = 10; GG *N* = 13).

a Significance determined using ANOVA for normally distributed means, Kruskal–Wallis test used for between group comparison of non-parametric variables and extra-sum of squares *F* test (for CCRC pooled best fit ET-1 data). There were no differences in between group baseline demographics in this vascular sub-study.

Notably, the selective ET_A_ receptor antagonist, BQ123, caused a parallel rightward shift of the CCRC with comparable pK_B_ values between groups AA, AG, and GG [pK_B_ values of 7.07 (±0.23), 7.79 (±0.35), and 7.41 (±0.26), respectively; *P* = 0.209; *Figure 4B*]. Zibotentan, a highly selective orally active ET_A_ receptor antagonist, attenuated the constrictor response to ET-1 with pK_B_ of 7.54 (95% CI 7.27–7.82), comparable to that of BQ123, pK_B_ 7.53 (95% CI 7.37–7.69).

Crucially, these studies confirmed that zibotentan produced a concentration-dependent inhibition of an established constrictor response to ET-1 and was still efficacious in subjects with G allele (*P* < 0.001; *Figure 4C*). *Figure 5* shows representative investigations from a female subject with few traditional cardiovascular risk factors but high-risk ET-1 enhancer genotype (GG).

**Take home figure:**
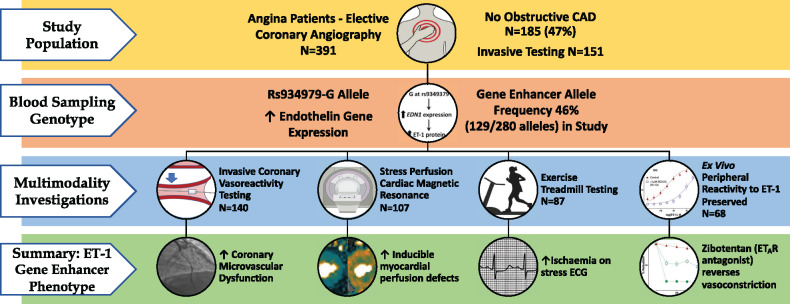
Study overview: endothelin-1 gene enhancer in microvascular angina. Three hundred and ninety-one patients with stable angina were prospectively enrolled without prior knowledge of coronary anatomy. One hundred and eighty-five (47%) had no obstructive coronary artery disease and thus eligible for invasive coronary vasoreactivity testing and further sub-studies. One hundred and fifty-one of 185 (82%) were able to undergo adjunctive invasive tests for coronary microvascular dysfunction. One hundred and nine (72%) subjects tested had evidence of coronary microvascular dysfunction. One hundred and forty subjects underwent genetic analysis for rs9349379-G allele with an allele frequency of 46% (129/280 alleles). The frequency of detrimental G alleles was higher than reference genome bank control subjects (46% vs. 39%; *P* = 0.013). Patients with rs9349379-G allele had higher serum endothelin-1 and over double the odds of coronary microvascular dysfunction (odds ratio 2.33, 95% confidence interval 1.10–4.96; *P* = 0.027). In addition, subjects were more likely to have impaired myocardial perfusion (*P* = 0.04) and exercise tolerance (−3.0 units in Duke Exercise Treadmill Score; *P* = 0.045). Peripheral small artery reactivity to endothelin-1 and affinity of ET_A_ receptor antagonists were preserved in the rs9349379-G allele group (*P* = 0.209). Crucially, zibotentan tested at clinically relevant concentrations, fully reversed an established endothelin-1 vasoconstriction, indicative of efficacy in conditions associated with vasospasm. This suggests that ET_A_ receptor antagonism in this group of patients may have therapeutic benefit.

## Discussion

We identify a novel genetic risk locus for CMD. Our study extends a report from the WISE investigators on genotype associations with arterial vasomotion.^13^ Our results support the hypothesis that dysregulation of the ET-1/ET_A_ receptor system underpins abnormalities in the coronary microcirculation leading to myocardial ischaemia. Firstly, rs9349379-G allele status is associated with higher serum ET-1 and the presence and extent of CMD in patients with angina but without obstructive coronary disease. Secondly, the genetic polymorphism associates with ischaemia testing using distinct, non-invasive modalities including exercise stress electrocardiography and stress perfusion CMR. Thirdly, we demonstrate in *ex vivo* human small peripheral resistance vessels that the ET_A_ vasoconstrictor response is not down-regulated in the presence of increases in endothelin gene expression and ET-1 activity in patients with the rs9349379-G allele. Finally, we provide proof-of-concept mechanistic data supporting a role for zibotentan, an orally active highly selective ET_A_ receptor antagonist, in reversing established ET-1-mediated vasoconstriction. These findings have potential clinical relevance since zibotentan is available for repositioning as a novel, disease-modifying therapy in this patient population. The results of our study support the rationale for the ‘Precision Medicine with Zibotentan in Microvascular Angina (PRIZE)’ trial involving gene testing for the SNP rs9349379 and linked therapy (ClinicalTrials.gov Identifier: NCT04097314).

### Endothelin dysregulation

Pre-clinical studies in experimental models of CMD implicate increased cardiac ET-1 production leading to endothelial dysfunction, enhanced vascular expression of rho-kinases, and reactive oxidant species such as superoxide and enhanced ET-1-mediated vasoconstriction.^38^ In patients with angina but no obstructive CAD, microvascular dysfunction is a systemic phenomenon characterized by peripheral endothelial dysfunction and enhanced peripheral small-vessel vasoconstriction.^31^^,^^39^ Further, impaired coronary microvascular function and the propensity to myocardial ischaemia may increase longer-term risk of major adverse cardiac events.^40^^,^^41^ Our study is distinct and builds on our prior vascular studies of ET-1 in microvascular angina as we used zibotentan which has more potential for clinical translation requiring future phase II studies.^31^ In addition, subjects were analysed by ET-1 rs9349379-G allele status rather than presence or absence of CMD. We observed that chronic exposure to increased circulating concentrations of ET-1, as reflected by rs9349379-G allele status, did not lead to down-regulation to ET_A_-mediated ET-1 vasoconstriction in patients with microvascular angina. The converse SNP (rs9349379-A) was recently found to be associated with spontaneous coronary artery dissection (SCAD) which typically occurs in patients without atherosclerosis.^21^ This finding is consistent with our work, particularly given that microvascular function is typically normal in SCAD.^42^

We showed that rs9349379-G allele was associated with higher serum ET-1 levels which is consistent with previous studies whereby the SNP associates with higher levels of ET-1 and its precursor (Big ET-1) in healthy subjects. Interestingly, the ET-1 plasma concentration in our INOCA population is comparable to ET-1 plasma concentrations in other conditions including pulmonary artery hypertension^43^ but lower than in other INOCA cohorts.^44^ We acknowledge that abluminal secretion of ET-1 away from endothelial cells towards underlying vascular smooth muscle means circulating concentrations of ET-1 are an imperfect measure of ET-1 activity in vascular tissues.^45^ Chronic elevation of circulating ET-1 may lead to adaptive down-regulation of its endogenous G-protein coupled receptors. This phenomenon has been described for ET_A_ receptors in mice in which the clearing ET_B_ receptor has been knocked out.^46^ Cardiovascular risk factors, including blood pressure, were not associated with rs9349379-G allele in our population, whereas an inverse associations have been observed in much larger populations.^17^ This is particularly interesting given its association with atherogenesis and CAD. It is thought that excess ET-1 effects healthy populations mediate hypotension via hypotension via ET_B_-induced nitric oxide and prostacyclin production, resultant vasodilation, diuresis, and natriuresis.^47^ Our study was underpowered to determine significant differences between baseline blood pressures which may also be confounded by treatment for hypertension.

Microvascular angina is a chronic, debilitating condition of unmet therapeutic need. Our vascular pharmacology findings indicate that despite a genetic predisposition to enhanced endothelin gene expression based on the rs9349379-G allele status, potentially leading to lifelong enhanced exposure to circulating concentrations of ET-1, the net effect on ET-1 response or sensitivity to ET_A_ antagonists was similar between the groups by rs9349379 allele status. The ET_A_ receptor may not be down-regulated in affected patients raising the potential for health gain by treatment with a selective ET_A_ receptor antagonist, such as zibotentan. Importantly, BQ123 fully blocked the constrictor responses in all of the groups. Our vascular pharmacology study was specifically focused on the relationships between the rs9349379-G allele status, ET-1 vasoactive responses, and ET_A_ receptor blockade. Patients with microvascular angina may have similar tissue responses to oral ET_A_ receptor blocker therapy—this important possibility merits further (NCT04097314).

In a mechanistic, randomized, controlled trial in patients with microvascular angina, Johnson and Gould^48^ reported that ET_A_ receptor antagonism increased (improved) the homogeneity of resting myocardial perfusion. Their study used cardiac positron emission tomography (PET) to quantify the homogeneity index (a visual notion of homogeneity derived from PET).^49^ Kaski *et al.*^50^ showed that patients with microvascular angina were exposed to increased circulating concentrations of ET-1 which in turn was associated with increased coronary vascular resistance and impaired coronary blood flow. Recently, Theuerle *et al.*^51^ have shown that plasma ET-1 is associated with invasive CMD in a 32 INOCA patients, however, the relationship was driven by elevated microvascular resistance and not impaired CFR.

### Limitations

We describe compelling mechanistic evidence for a functional SNP being linked to CMD. We have followed accepted guidelines for CMD classifications, but it is recognized there are caveats with any classification system and acknowledge these are also relevant to this study. Firstly, we adopted binary cut-offs for the IDP test. It is possible that indeterminate (grey-zone or borderline) test results may have misclassified some patients. Furthermore, patients with CMD were heterogeneous and we aggregated patients with different types of microvascular dysfunction, e.g. impaired flow reserve, increased microvascular resistance, abnormal ACh response. Nonetheless, the vascular phenotype of affected patients was of coronary vascular dysfunction based on consensus guidelines for abnormal coronary microvascular response during systemic adenosine, an abnormal vasomotor response to intracoronary ACh, or both.^6^ In support of this approach, we observed a strong linear relationship between CMD and non-invasive ischaemia testing on the exercise treadmill (*Figure 3F*). In addition, heterogeneity is the rule rather than exception when considering many similar cardiovascular disorders, for example heart failure with preserved ejection fraction.^52^ Our stratified sensitivity analysis by CMD type, i.e. structural microvascular disease (i.e. raised IMR) and impaired vasodilator reserve (reduced CFR) (*Table 2*), lend further support to the design of our translational study. Secondly, not all patients underwent treadmill exercise testing. The tests were indicated as part of standard care and clinical, rather than core laboratory, reports were available for analysis. Nevertheless, they were performed according to the Bruce protocol and the results were determined in a standardized manner, blinded to rs9349379 allele status. Treadmill exercise testing is an imperfect measure of ischaemia and hence it is plausible that the known association of the rs9349379-G allele with epicardial CAD is a confounding factor. Gould and Johnson^53^ recently highlighted how flush ostial branch vessel occlusion may account for ischaemia despite a visual ‘normal’ angiogram without stenosis. On the other hand, the DTS has a mature associated literature with proven utility in CMD patients.^54^^,^^55^ The relatively small sample size and possibility of unmeasured baseline differences increases the possibility of Type I error. Thirdly, we administered intra-arterial doses of short acting GTN (100–200 μg) to facilitate procedure safety relating to transradial access, coronary arteriography, and invasive coronary vasoreactivity testing. Theoretically, GTN may affect the vascular responses to ACh; however, the half-life of GTN is around 2 min. Hence, after 10 min, only 3% of the GTN dose is bioavailable and we think the potential for confounding and a false negative test for microvascular vasospasm is unlikely. Conversely, a positive ACh test confounds assessment of true resting flow and may lead to falsely lowered CFR and hence we support ACh testing after adenosine assessment. Finally, we compared the allele prevalence within our cohort from Scotland with a pooled multicentre contemporary medical genome reference group of controls. Our study would have been strengthened by a control comparator group from the same area and ethnic background as our subjects. Further, although the SNP did not fulfil the Hardy–Weinberg equilibrium for the population as a whole, the control group from this study without CMD was consistent with the equilibrium (χ^2^ 2.99, *P* = 0.084). It is plausible that HW was not met in the CMD group due to its association with the rs9349379-G allele of interest. This study is a cross-sectional analysis of a single genetic locus and provides associative findings of clinical interest but may overlook other important genetic risk determinants.

### Clinical translation

These observations hypothesis generating particularly given the small sample size and heterogeneous patient population. The findings require external validation in other CMD cohorts whilst future work in populations from different regions would provide helpful context.

Overall, our study supports the case for selective ET_A_ blockade distinct from ET_B_ modulation in patients with microvascular disease in the heart. Oral ET_A_-selective blockade has therapeutic potential by attenuating the propensity to microvascular vasospasm, increasing coronary blood flow, and further improving coronary endothelial function through NO-mediated release.^56^ Zibotentan is one compound that holds promise as the most ET_A_ selective of all orally active ET_A_ receptor antagonists, which makes it particularly suited to use in microvascular angina. A targeted approach using selective ET_A_ receptor antagonist therapy in patients based on genotype is being assessed in the PRIZE trial (NCT04097314).

## Conclusion

We identified a genetic risk locus for CMD. The common genetic polymorphism (SNP rs9349379-G allele) was associated with higher ET-1 and both invasive CMD and non-invasive tests for ischaemia in subjects with angina but no obstructive CAD. Mechanistic *ex* *vivo* studies confirmed subjects with this functional allele have preserved response to ET_A_ receptor blockade. Zibotentan, an orally active ET_A_ receptor antagonist, reversed an established ET-1-mediated vasoconstriction. This study offers hope for angina patients although future trials are needed to determine whether CMD represents a potential new disease subtype for ET_A_ antagonist therapy.

## Supplementary Material

ehz915_Supplementary_Data

## Notes

**See page 3252 for the editorial comment on this article (doi: 10.1093/eurheartj/ehz954)**
