# Supplementary material for: Genetic dysregulation of endothelin-1 is implicated in coronary microvascular dysfunction
Source: Eur Heart J. 2020 Jan 23;41(34):3239–52. doi: 10.1093/eurheartj/ehz915 (PMC7557475; doi:10.1093/eurheartj/ehz915)
Supplement: ehz915_Supplementary_Data [file ehz915_supplementary_data.docx]

Supplementary Information (Appendix)

**Genetic dysregulation of endothelin-1 is implicated in coronary microvascular dysfunction**

This appendix has been provided by the authors to give readers additional information about the study methods.

Table of Contents

[1. Supplementary Figure 1 – ET-1 in CMD subjects 3](#_Toc24395806)

[2. Supplementary Figure 2 – Angina on treadmill testing 4](#_Toc24395807)

[3. Supplementary Figure 3: Competition Binding Curve for zibotentan 5](#_Toc24395808)

[4. Supplementary Table 1. Genotype as multivariable predictor of CMD 6](#_Toc24395809)

[*5.* Supplemental Methods: Measurement of coronary vascular function *in vivo* 7](#_Toc24395810)

[6. Angiographic analysis and quantitative coronary angiography (QCA) 9](#_Toc24395811)

[7. Definitions: coronary microvascular dysfunction 10](#_Toc24395812)

[8. Blood and tissue analysis 11](#_Toc24395813)

[9. DNA extraction and genotyping 13](#_Toc24395814)

[10. Cardiac magnetic resonance imaging and ischaemia testing protocol 14](#_Toc24395815)

[11. Peripheral vascular function assessment 17](#_Toc24395816)

[12. Statistical Considerations 21](#_Toc24395817)

[13. References 22](#_Toc24395818)

# Supplementary Figure 1 – ET-1 in CMD subjects

A: Serum ET-1was higher in patients with coronary microvascular dysfunction than normal control subjects [P=0.040]. ET-1 was adjusted for baseline confounders in regression model outlined in study methods. B: Normalized serum ET-1 (Log transformed) showed a trend towards significance with increasing peptide linearly with each G allele [P linear trend = 0.131].

# Supplementary Figure 2 – Angina on treadmill testing

Exercise treadmill testing of angina patients without obstructive CAD by genotype group according to presence or absence of angina during exercise. There was a significant linear association of angina status with rs9349379 genotype (P=0.019)

# Supplementary Figure 3: Competition Binding Curve for zibotentan

Competition Binding Curve for zibotentan in human left ventricle confirms selectivity for the ET_A_ receptor

# Supplementary Table 1. Genotype as multivariable predictor of CMD

|  | B | OR | Lower CI | Upper CI | P-value |
| --- | --- | --- | --- | --- | --- |
| Genotype (per G allele) | 0.84 | 2.31 | 1.08 | 4.91 | 0.030 |
| ASSIGN | 0.00 | 1.00 | 0.98 | 1.02 | 0.970 |
| Past CV event | 0.23 | 1.26 | 0.51 | 3.15 | 0.619 |

Multivariable predictors of any CMD (N=109) with three baseline factors in model listed above. The fitted regression model showed moderate discrimination potential with an AUC of 0.647 (95% CI 0.544 – 0.750; P=0.007). ASSIGN refers to estimated 10-year risk of cardiovascular events incorporating social deprivation in a validated score within our geographical area.^1^

# Supplemental Methods: Measurement of coronary vascular function *in vivo*

We used an interventional diagnostic protocol that combined guidewire-based direct measurement of coronary vascular function followed by pharmacological vasoreactivity testing. Specifically, the procedure included a guidewire-based measurement of coronary vascular function (FFR, coronary flow reserve [CFR], and the index of microvascular resistance [IMR]) followed by pharmacological vasoreactivity testing with acetylcholine (ACh) and glyceryl trinitrate (GTN) and has been previously described.**^2, 3^**

In brief, an intravenous infusion of adenosine (140 μg·kg^−1^·min^−1^) was administered via a large peripheral vein to induce steady-state maximal hyperaemia. A pressure-temperature sensitive guidewire was placed into the distal third of a major epicardial coronary artery (typically the left anterior descending [LAD]). The myocardial FFR was calculated by the ratio of mean distal coronary pressure to mean aortic pressure at maximal hyperaemia. A FFR ≤0.80 was taken as abnormal and indicative of flow-limiting coronary artery disease.^4^ CFR was calculated using thermodilution as resting mean transit time divided by hyperaemic mean transit time.^5^ A CFR <2.0 was defined as abnormal representing impaired vasodilator reserve.^6^ The IMR was calculated as the product of mean hyperaemic transit time and mean distal coronary pressure at hyperaemia.^7^ An IMR >25 was defined as abnormal and indicative of increased microvascular resistance.^8^ These invasive parameters were simultaneously derived in real-time using dedicated software (Coroventis, Uppsala, Sweden). We assessed endothelium-dependent coronary vasomotor function using intra-coronary infusions of ACh via the guiding catheter at concentrations of 0.182, 1.82, and 18.2 µg/mL (10^-6^, 10^-5^, and 10^-4^ mol/L, respectively) at 1 mL/min for 2 minutes via a mechanical infusion pump.^9^ We then immediately performed provocation testing for microvascular or epicardial coronary artery spasm using a 100μg bolus of ACh (5.5 mL of 10^-4^ mol/L over 20 seconds – reduced to 50μg for the RCA). In order to assess non-endothelial dependent vasodilatation, 300 µg of GTN was administered by manual intra-coronary bolus injection.

# Angiographic analysis and quantitative coronary angiography (QCA)

Quantitative coronary analysis of the target coronary artery was performed using computer-assisted angiographic analysis (QAngio XA7.3, Medis, Leiden, Netherlands) by a trained cardiologist. Fluoroscopic images from two angles at least 30° apart were acquired. The coronary artery (typically left anterior descending artery) measurements were performed in the region where the greatest change had occurred during coronary reactivity testing.^10^ End-diastolic cine frames that best show the segment were selected, and calibration of the video and cine images was performed. Coronary artery diameter change (% from baseline) was measured in response to both ACh and glyceryl trinitrate. Severe endothelial dysfunction was defined by ≥20% luminal constriction during ACh infusion (up to 10^−4^M); this finding implies significant reduction in coronary artery blood flow with prognostic implications when compared with patients whose arteries were <20% constricted.^11^ Coronary artery disease severity was assessed using the Gensini score.^12^A second trained observer (PM) performed QCA on a consecutive sample of 10% of cases, with high concordance for measurements of percentage lumen diameter vasoconstriction during ACh vasospasm assessment (intraclass correlation coefficient for average measures 0.96; 95% CI 0.88-0.99; p<0.001) and Gensini angiographic score (intraclass correlation coefficient for average measures 0.99; 95% CI 0.96-1.00; p<0.001).

# Definitions: coronary microvascular dysfunction

We defined CMD using invasive coronary function testing and the Coronary Vasomotion Disorders International Study Group (COVADIS) diagnostic criteria.^13^ These physiological criteria included raised IMR, abnormal coronary vasodilator capacity (CFR) and/or microvascular spasm during ACh provocation (reproduction of angina symptoms, ischaemic ECG changes (≥1mm ST segment deviation), but < 90% epicardial spasm during ACh testing).^14^ FFR was measured to rule-out flow limiting coronary artery disease as an alternative explanation for myocardial ischaemia. Therefore, all participants had an FFR >0.8 in the target coronary artery and participants with an FFR ≤0.80 were excluded.

# Blood and tissue analysis

Serum ET-1 was determined using blood obtained on the day of coronary function testing (Quantikine ® ELISA, R&D Systems® Europe, Abington [UK]). Blood was obtained from participants following an overnight fast in a recumbent position.

*Ex vivo* pharmacological assessment of peripheral vascular function was performed on patients who volunteered to undergo a gluteal skin fat biopsy within 4 weeks of the invasive coronary function assessment. The biopsy was obtained under sterile conditions using local anaesthesia with lidocaine (2%). Arterioles (< 400µm) were carefully dissected from fresh biopsies using a light microscope. 2mm length arterioles were mounted on 40-μm stainless steel wires for isometric myography in multi-channel myograph chambers (DMT, Denmark) filled with physiological saline solution. Isometric tension recordings followed-on directly using the technique of wire myography to study small peripheral resistance arteries with paired cumulative concentration response curves (CCRCs) to ET-1 in the presence or absence of an ET_A_ receptor antagonist, either BQ123 or zibotentan. The detailed methods are described in the study appendix. The peripheral vascular sensitivity to ET-1 (pEC_50_) and maximum vasoconstriction to ET-1 (E_max_) were determined.

For the antagonist studies the affinity (K_B_) of BQ123 was first determined in paired vessels from individuals and calculated using Schild regression. The pK_B_ (-log_10_ K_B_) values were compared between each genotype as an indicator of whether or not patients of different genotypes are likely to respond equally well to an ET_A_ antagonist used clinically. A final series of experiments involved paired vessel experiments using ET-1 CCRCs in the presence and absence of a highly selective ET_A_ receptor antagonist, zibotentan to determine a pK_B_ value. More importantly the aim of these experiments was also to evaluate whether zibotentan could reverse an established ET-1 mediated vessel constriction.

# DNA extraction and genotyping

Buffy coat was extracted from the whole blood of patients after centrifugation at 10,000g) to isolate genomic DNA using The PureLink® Genomic DNA Mini Kit (Invitrogen™). The samples were added to the lysis/binding buffer and digested with Proteinase K and RNase A for a minimum of 10 minutes at 50^o^C. The samples were then brought to room temperature, followed by the addition of absolute ethanol. This was applied to the PureLink® Spin column and centrifuged, followed by subsequent washing and elution using Tris EDTA buffer. The quantity and quality of the DNA extracted was determined using NanoDrop™ Lite Spectrophotometer (ThermoFisher Scientific™).

To determine the genotype for rs9349379, the probes TaqMan® SNP Genotyping Assay ID C___1756707_10, with the context Sequence **[VIC/FAM]**: TCTATGCCCTTGAGATCATATAAAA**[A/G**]TAGCTTAAAATCATTGGCCATAGTT (Applied Biosystems™). DNA concentration of 5ng/uL was used with the TaqMan® probes and TaqMan® Universal Master Mix II, no UNG (Applied Biosystems™) to a total reaction volume of 5uL. This was amplified and read using QuantStudio™ 12K Flex (Applied Biosystems™) to determine the genotype.

# Cardiac magnetic resonance imaging and ischaemia testing protocol

Patients were invited to undergo quantitative perfusion cardiac magnetic resonance (CMR) imaging at 1.5 Tesla using pharmacological stress testing with intravenous adenosine (140 µg/kg/min) within 6 weeks of the index coronary angiogram. CMR studies were performed using a standardized CMR protocol (Siemens MAGNETOM Avanto, Erlangen, Germany). Qualitative review for an inducible subendocardial perfusion defect consistent with microvascular dysfunction was independently performed by two cardiologists blinded to patient genotype. Quantitative measurement of the myocardial perfusion reserve ratio using a novel pixel-mapping technique was also performed (Supplementary information). Treadmill exercise stress electrocardiography using the Bruce protocol was analysed from the sub-group of patients who had been pre-selected for this procedure on clinical grounds prior to invasive coronary angiography. The exercise ECG parameters including (1) exercise duration and (2) the Duke Treadmill Score^15^ were analysed by a cardiology researcher (EY) blinded to genotype and invasive physiology.

All subjects were asked to abstain from caffeine-containing beverages or foodstuffs for 24 hours, and vasoactive medications for 48 hours prior to the CMR examination. All scan acquisitions were spatially co-registered. All CMR analyses were performed by a blinded analyst with Level 3 EACVI accreditation.

### Myocardial perfusion

Stress and rest first-pass perfusion imaging were performed using an echo planar imaging (EPI) dual-sequence investigational perfusion method, which consists of a low resolution arterial input function (AIF) image, followed by three short axis (base, mid, apex) myocardial images during each R-R interval.^16-18^ First-pass perfusion images were obtained in 3 LV short-axis slices and one long-axis slice. Vasodilator stress was achieved with adenosine infusion 140-210 µg/kg/min for 3 minutes. Resting first-pass perfusion was performed at least 10 minutes later.

The raw stress and rest perfusion images were qualitatively assessed for inducible or fixed perfusion defects. The perfusion was classified as either normal, abnormal, or equivocal. If a perfusion defect was present, it was reported as having and epicardial, microvascular or equivocal pattern. Normal myocardial perfusion was depicted by homogeneous first pass perfusion as revealed by dynamic first pass perfusion imaging and the pixel maps. An inducible perfusion defect on dynamic first pass imaging during adenosine hyperaemia was reflected by a relative reduction in myocardial signal intensity notably in the sub-endocardium extending radially. The onset of the defect would occur with the arrival of the gadolinium contrast media in the left ventricular blood pool, it would persist beyond peak myocardial enhancement for 5 or more R-R intervals, and regresses over time towards the sub-endocardium. The defect would be present during stress but not resting conditions. The defect may conform to the myocardial blood supply of an epicardial coronary artery in a transmural distribution, or if the defect is primarily due to microvascular disease, the defect may be circumferential and restricted to the sub-endocardium. The perfusion defect should be 2 or more pixels wide.^19^ An equivocal perfusion abnormality would meet some but not all of these criteria, raising a suspicion of a perfusion abnormality but not clearly diagnostic. Perfusion defects were reported on a segmental basis according to the American Heart Association 16-segment model.^20^ Dark banding artefact was adjudicated based on standardised criteria.^19^

Pixel-wise perfusion maps were generated and analysed to derive fully quantitative MBF estimates on a pixel-wise basis in ml/g/min of myocardium. The pixel-wise perfusion method used a series of automated post-processing steps on the raw Digital Imaging and Communications in Medicine (DICOM) images to generate fully quantitative pixel maps. The pixel-wise time-signal intensity curves were then quantified using model-constrained Fermi deconvolution.^21-23^

### Extra-cardiac anatomy and LV volumes, function and mass

Fast gradient echo ‘white-blood’ images in the axial, coronal and sagittal planes were obtained, and were qualitatively assessed for extra-cardiac anatomy and pathology, and clinically-relevant incidental findings.

Steady-state free procession (SSFP) ‘cine’ imaging using a trueFISP sequence (multi-slice single-shot breath-hold true fast imaging) was performed in the 3 long-axis planes and short axis cine ‘stack’ for assessment of LV volumes, function and mass.

### Myocardial tissue characterisation

Native T1 mapping was performed using a modified look-locker inversion-recovery (MOLLI) investigational prototype sequence. Images were obtained in three short-axis slices (base, mid, apex). T1 mapping was performed pre- and post-gadolinium contrast to assess the myocardial native T1 relaxation time and estimate the myocardial extracellular volume (ECV) in both the mid-septum and globally.^24^

Late gadolinium enhancement imaging was performed using a segmented phase-sensitive inversion recovery (PSIR) turbo fast low-angle shot imaging sequence.^25^ Images were obtained in the three long-axis planes and short-axis images covering the entire left ventricle. The pattern and burden of hyper-enhancement was both qualitatively and quantitatively assessed.

# Peripheral vascular function assessment

**Competition binding study in human heart to confirm ET_A_ selectivity of zibotentan**

Initial reports on the pharmacology of zibotentan demonstrated that the compound had high affinity for the human cloned ET_A_ receptor (21 nmol/L) and no detectable affinity at human cloned ET_B_ receptors.^26^ We have confirmed ET_A_ selectivity in human heart, a tissue that expresses both ET_A_ and ET_B_ receptors.

Briefly, human heart was collected with informed patient consent and local ethical approval. Competition binding experiments (n=3) were performed in cryostat-cut frozen heart sections (10μm) with [^125^I]ET-1 (0.1nM) in the presence of increasing concentrations of zibotentan (2 pmol/L-100 μmol/L). Non-specific binding was determined using 1μol/L ET-1. Data were analysed to obtain affinity, pKi (the –log_10_of the equilibrium dissociation constant K_i_ determined in a competition binding assay), for zibotentan at ET_A_ and ET_B_ receptors using GraphPad Prism 6. Zibotentan competed in a biphasic manner (Supplementary Figure 2) resulting in a pK_i_±SEM for the ET_A_ receptor of 9.88±0.13 and for the ET_B_ receptor of 4.02±0.04 indicating a >720,000 fold selectivity for the human ET_A_ compared to the human ET_B_ receptor in heart.

Experiments were designed to investigate whether patients with the SNP G allele, who had therefore been exposed to higher levels of endogenous endothelin-1 (ET-1), exhibited a change in responsiveness of vascular smooth ET_A_ receptors assessed *in vitro*. Initial studies determined whether there was evidence of endothelial dysfunction, indicated by a change in either potency or maximum response to the endothelium-dependent vasodilator acetylcholine (Ach) or whether the response to ET-1 was altered and if changes for either compound correlated to whether individuals expressed the SNP G allele or not. Finally, it was important to establish that, whatever the underlying genotype, responses to ET-1 could be blocked by ET_A_ antagonists. Two antagonists were tested. The very well characterised peptide antagonist BQ123 and importantly the orally active, highly selective ET_A_ antagonist zibotentan that has the potential to be rapidly repurposed for clinical use in this patient group.

## Preparation of small resistance arteries

Vessels were dissected from a gluteal skin fat biopsy (approximately 3 x 2 x 2 cm) performed within 4 weeks of coronary angiography. Vasoactive medications were withheld for at least 24 hours prior to the surgical biopsy. Small resistance arterioles (normalised diameter <400 μm) were studied in a Mulvany-Halpern 4-channel wire myograph (Danish Myotech, Aarhus, Denmark) with isometric tension recordings made as previously described.^27^

## Experimental Protocol

After a standard normalisation and start-up protocol involving repeated washes with high potassium chloride solution (62.5 mmol/L KPSS), the arterioles were pre-constricted with the thromboxane-A2 analogue, U46619 (0.1 μmol/L). Previous work on human resistance arteries support its application in myography due to its consistent vasoconstriction with a steady plateau from which to assess arteriolar relaxation. Blood vessels with no responses were discarded. For viable tissue the integrity of the endothelium was determined by constructing a cumulative concentration-response curve (CCRC) to ACh (1n mol/L - 1µ mol/L). CCRCs were then obtained to the vasoconstrictor peptide ET-1(1 p mol/L - 1 μmol/L).

For relaxation data, responses to ACh were expressed as the percentage reversal of the constrictor response to U46619 (100nmol/L). Data for ET-1 were normalised as a percentage of the mean response to the last two responses to 62.5mol/L potassium chloride obtained during the set up procedure. CCRCs were fitted using four‐parameter, non‐linear regression curve fitting in Prism 7.0 (GraphPad Inc, La Jolla, CA, USA) to obtain values of potency (expressed as the pEC_50_, that is the –log_10_ of the EC_50_ (the concentration producing half-maximal response)) and maximum response (E_max_) for both agonists. Derived parameters were compared for patients with or without the SNP G allele.

For antagonist studies paired tissues from patients were used to construct CCRCs to ET-1 in either the absence (control) or presence of 1 µmol/L BQ123 (N=27) or 1µmol/L zibotentan (N=8). Data were expressed as % KPSS as described above and paired CCRCs were then analysed using the Gaddum/Schild EC_50_ equation (GraphPad Prism). The Hill and Schild slopes were constrained to 1 and therefore antagonist affinity was given as the pK_B_ (the –log_10_ of the equilibrium dissociation constant). pK_B_ values for BQ123 derived from individuals with different SNP alleles were compared.

Finally, vessels from some individuals were preconstricted with ET-1 and the constrictor response allowed to stabilise before addition of either no antagonist (time matched control ) or 0.1µmol/L or 1µmol/L zibotentan was added. The contraction to ET-1 was monitored over the next 90 minutes to determine the extent of reversal by zibotentan over time.

# Statistical Considerations

Pharmacology definitions: potency (pEC50) is the –log10 of the concentration of a drug that gives half-maximal response; Efficacy is given as Emax, the % contraction to KPSS; pKB is the –log10 of the antagonist affinity (KB the equilibrium dissociation constant of the antagonist for the ETA receptor). For wire myography, we analysed pKB between three groups using one-way ANOVA and adopted a two-tailed model with alpha of 0.05 and beta of 0.2 giving a planned power of 80%. An estimated effect size (f) of 0.544 was determined using estimated group mean pKB values of 7.7, 7.6 and 7.5 respectively (SD of 0.15). A minimum sample size of 36 subjects was determined using G*Power 3.1 (University of Melbourne, Parkville VIC, Australia).

*References*

1. Woodward M, Brindle P, Tunstall-Pedoe H. Adding social deprivation and family history to cardiovascular risk assessment: the ASSIGN score from the Scottish Heart Health Extended Cohort (SHHEC). Heart 2007;**93**(2):172-6.

2. Ford TJ, Corcoran D, Oldroyd KG, McEntegart M, Rocchiccioli P, Watkins S, Brooksbank K, Padmanabhan S, Sattar N, Briggs A, McConnachie A, Touyz R, Berry C. Rationale and design of the British Heart Foundation (BHF) Coronary Microvascular Angina (CorMicA) stratified medicine clinical trial. Am Heart J 2018;**201**:86-94.

3. Ford TJ, Stanley B, Good R, Rocchiccioli P, McEntegart M, Watkins S, Eteiba H, Shaukat A, Lindsay M, Robertson K, Hood S, McGeoch R, McDade R, Yii E, Sidik N, McCartney P, Corcoran D, Collison D, Rush C, McConnachie A, Touyz RM, Oldroyd KG, Berry C. Stratified Medical Therapy Using Invasive Coronary Function Testing In Angina: CorMicA Trial. J Am Coll Cardiol 2018.

4. De Bruyne B, Baudhuin T, Melin JA, Pijls NH, Sys SU, Bol A, Paulus WJ, Heyndrickx GR, Wijns W. Coronary flow reserve calculated from pressure measurements in humans. Validation with positron emission tomography. Circulation 1994;**89**(3):1013-22.

5. Pijls NHJ. Coronary Thermodilution to Assess Flow Reserve: Validation in Humans. Circulation 2002;**105**(21):2482-2486.

6. Murthy VL, Naya M, Taqueti VR, Foster CR, Gaber M, Hainer J, Dorbala S, Blankstein R, Rimoldi O, Camici PG, Di Carli MF. Effects of sex on coronary microvascular dysfunction and cardiac outcomes. Circulation 2014;**129**(24):2518-27.

7. Fearon WF, Balsam LB, Farouque HM, Caffarelli AD, Robbins RC, Fitzgerald PJ, Yock PG, Yeung AC. Novel index for invasively assessing the coronary microcirculation. Circulation 2003;**107**(25):3129-32.

8. Lee BK, Lim HS, Fearon WF, Yong AS, Yamada R, Tanaka S, Lee DP, Yeung AC, Tremmel JA. Invasive evaluation of patients with angina in the absence of obstructive coronary artery disease. Circulation 2015;**131**(12):1054-60.

9. Lerman A, Holmes DR, Bell MR, Garratt KN, Nishimura RA, Burnett JC. Endothelin in Coronary Endothelial Dysfunction and Early Atherosclerosis in Humans. Circulation 1995;**92**(9):2426-2431.

10. Reriani M, Raichlin E, Prasad A, Mathew V, Pumper GM, Nelson RE, Lennon R, Rihal C, Lerman LO, Lerman A. Long-term administration of endothelin receptor antagonist improves coronary endothelial function in patients with early atherosclerosis. Circulation 2010;**122**(10):958-66.

11. Suwaidi JA, Hamasaki S, Higano ST, Nishimura RA, Holmes DR, Jr., Lerman A. Long-term follow-up of patients with mild coronary artery disease and endothelial dysfunction. Circulation 2000;**101**(9):948-54.

12. Gensini GG. A more meaningful scoring system for determining the severity of coronary heart disease. Am J Cardiol 1983;**51**(3):606.

13. Rose G, McCartney P, Reid DD. Self-administration of a questionnaire on chest pain and intermittent claudication. Br J Prev Soc Med 1977;**31**(1):42-8.

14. Beltrame JF, Crea F, Kaski JC, Ogawa H, Ong P, Sechtem U, Shimokawa H, Bairey Merz CN, Coronary Vasomotion Disorders International Study G. International standardization of diagnostic criteria for vasospastic angina. Eur Heart J 2017;**38**(33):2565-2568.

15. Mark DB, Shaw L, Harrell FE, Jr., Hlatky MA, Lee KL, Bengtson JR, McCants CB, Califf RM, Pryor DB. Prognostic value of a treadmill exercise score in outpatients with suspected coronary artery disease. N Engl J Med 1991;**325**(12):849-53.

16. Miller CA, Hsu LY, Ta A, Conn H, Winkler S, Arai AE. Quantitative pixel-wise measurement of myocardial blood flow: the impact of surface coil-related field inhomogeneity and a comparison of methods for its correction. Journal of cardiovascular magnetic resonance : official journal of the Society for Cardiovascular Magnetic Resonance 2015;**17**:11.

17. Benovoy M, Jacobs M, Cheriet F, Dahdah N, Arai AE, Hsu LY. Robust universal nonrigid motion correction framework for first-pass cardiac MR perfusion imaging. J Magn Reson Imaging 2017;**46**(4):1060-1072.

18. Zierler KL. Equations for Measuring Blood Flow by External Monitoring of Radioisotopes. Circ Res 1965;**16**:309-21.

19. Schulz-Menger J, Bluemke DA, Bremerich J, Flamm SD, Fogel MA, Friedrich MG, Kim RJ, von Knobelsdorff-Brenkenhoff F, Kramer CM, Pennell DJ, Plein S, Nagel E. Standardized image interpretation and post processing in cardiovascular magnetic resonance: Society for Cardiovascular Magnetic Resonance (SCMR) board of trustees task force on standardized post processing. J Cardiovasc Magn Reson 2013;**15**:35.

20. Cerqueira MD, Weissman NJ, Dilsizian V, Jacobs AK, Kaul S, Laskey WK, Pennell DJ, Rumberger JA, Ryan T, Verani MS, American Heart Association Writing Group on Myocardial S, Registration for Cardiac I. Standardized myocardial segmentation and nomenclature for tomographic imaging of the heart. A statement for healthcare professionals from the Cardiac Imaging Committee of the Council on Clinical Cardiology of the American Heart Association. Circulation 2002;**105**(4):539-42.

21. Jerosch-Herold M, Wilke N, Stillman AE. Magnetic resonance quantification of the myocardial perfusion reserve with a Fermi function model for constrained deconvolution. Med Phys 1998;**25**(1):73-84.

22. Axel L. Tissue mean transit time from dynamic computed tomography by a simple deconvolution technique. Invest Radiol 1983;**18**(1):94-9.

23. Hsu LY, Groves DW, Aletras AH, Kellman P, Arai AE. A quantitative pixel-wise measurement of myocardial blood flow by contrast-enhanced first-pass CMR perfusion imaging: microsphere validation in dogs and feasibility study in humans. JACC Cardiovascular imaging 2012;**5**(2):154-66.

24. Moon JC, Messroghli DR, Kellman P, Piechnik SK, Robson MD, Ugander M, Gatehouse PD, Arai AE, Friedrich MG, Neubauer S, Schulz-Menger J, Schelbert EB, Society for Cardiovascular Magnetic Resonance I, Cardiovascular Magnetic Resonance Working Group of the European Society of C. Myocardial T1 mapping and extracellular volume quantification: a Society for Cardiovascular Magnetic Resonance (SCMR) and CMR Working Group of the European Society of Cardiology consensus statement. Journal of cardiovascular magnetic resonance : official journal of the Society for Cardiovascular Magnetic Resonance 2013;**15**:92.

25. Kellman P, Arai AE, McVeigh ER, Aletras AH. Phase-sensitive inversion recovery for detecting myocardial infarction using gadolinium-delayed hyperenhancement. Magn Reson Med 2002;**47**(2):372-83.

26. Morris CD, Rose A, Curwen J, Hughes AM, Wilson DJ, Webb DJ. Specific inhibition of the endothelin A receptor with ZD4054: clinical and pre-clinical evidence. Br J Cancer 2005;**92**(12):2148-52.

27. Hillier C, Berry C, Petrie MC, O'Dwyer PJ, Hamilton C, Brown A, McMurray J. Effects of urotensin II in human arteries and veins of varying caliber. Circulation 2001;**103**(10):1378-81.
